# A Rapid and Versatile Colorimetric Sensor for the Visual Detection of Zinc Ions in Urine and Drinking Water

**DOI:** 10.3390/s26061926

**Published:** 2026-03-19

**Authors:** Thea Serra, Fabio Di Nardo, Simone Cavalera, Valentina Testa, Stefano Bertinetti, Claudio Baggiani, Daniele Amparore, Sabrina De Cillis, Sergio Occhipinti, Laura Anfossi

**Affiliations:** 1 Department of Chemistry, University of Turin, 10125 Turin, Italy; thea.serra@unito.it (T.S.); simone.cavalera@unito.it (S.C.); v.testa@unito.it (V.T.); stefano.bertinetti@unito.it (S.B.); claudio.baggiani@unito.it (C.B.); laura.anfossi@unito.it (L.A.); 2 Department of Oncology, University of Turin, 10043 Orbassano, Italy; daniele.amparore@unito.it (D.A.); sabrinatitti.decillis@unito.it (S.D.C.); 3 Department of Molecular Biotechnologies and Health Sciences, University of Turin, 10126 Turin, Italy; occhipinti@nibbiotec.com; 4 NIB Biotec Srl, Via Candido Viberti 4, 10141 Turin, Italy

**Keywords:** paper-based, colorimetric sensor, zinc detection, drinking water, urine, prostate cancer

## Abstract

The rapid detection of zinc in different aqueous matrices is very relevant. For example, a Zn^2+^ level above ca. 50 µM affects drinking water quality, while levels below ca. 25 µM in urine are related to higher probability of prostate cancer. Herein, a simple and rapid qualitative colorimetric sensor for the detection of zinc ions in aqueous samples is developed. The sensor exploits the reaction between 1,5-diphenylthiocarbazone and Zn^2+^ to form colored chelates whose color changes with increasing Zn^2+^ concentration. The chelating agent has been immobilized in a dried form on various cellulose- and synthetic-based materials to obtain a sensor that can be used for in situ analysis. The procedure to obtain the colorimetric device is easy and straightforward. Moreover, it requires neither specialized personnel to perform the analysis nor specialized personnel for the interpretation of the analytical results. The analysis requires only 20 µL of sample, and a reliable colorimetric output is obtained within 10 min and is stable up to 30 min. The sensor allows Zn^2+^ visual detection in drinking water and urine without any sample pre-treatment with excellent efficiency and repeatability. Considering the ability to distinguish between Zn^2+^ concentrations equal to 0.5 and 2× the cut-off level, the sensor showed sensitivity and specificity of 100% for fortified tap water analysis and 100% sensitivity and 88.9% specificity for urine samples. The almost-perfect concordance with the reference atomic absorption spectrometer and the 94.1% accuracy demonstrated the sensor’s excellent potential to be applied for selective qualitative Zn^2+^ detection in real-life situations.

## 1. Introduction

Zinc (Zn) is a crucial trace element, essential for various biological processes and environmental health [1]. In water, zinc concentrations can be elevated by industrial discharge and urban runoff, making it imperative to monitor and control its levels to prevent ecological harm and ensure water safety. Luckily, no health-based guideline value is in force for Zn in drinking water. However, based on aesthetic considerations, Zn concentration should be less than 3 mg/L (ca. 50 µM) to avoid astringent taste [2]. At the same time, zinc levels in biological fluids are also related to different pathologies [3], and recently, it has also been demonstrated that the Zn level in urine is correlated with prostate cancer [4,5].

Current detection methods for zinc include atomic absorption spectroscopy (AAS) and inductively coupled plasma mass spectrometry (ICP-MS) as the gold standard [6]. These methods are highly sensitive and accurate but have significant drawbacks, including high costs, the need for sophisticated equipment, and extensive sample preparation, which limit their use for field applications and real-time monitoring [7,8,9]. Other detection methods like electrochemistry, spectrophotometry and spectrofluorimetry have been successfully applied, but they still share the common limitations of gold-standard methods [7,8,9,10].

Rapid and in situ detection techniques may overcome the limitations of traditional zinc detection methods. These analytical techniques, often referred to as Point-of-Need (PON) tests, are particularly advantageous as they are portable, cost-effective, and capable of providing immediate results [11]. Among them, μPADs offer significant potential for PON detection of metal ions, especially when exploiting colorimetric detection methods that may not require an external reader [12,13]. These systems provide benefits like reduced reagent use, greater ease of use, and shorter analysis time, though they may have lower specificity compared to traditional methods [12,13].

Many colorimetric μPADs have been developed for the detection of metal ions in different matrices, and only a few have been reported for the detection of zinc ions in aqueous matrices [14]. Usually, these systems operate using dyes that can form complexes with zinc ions, producing a color change. For example, Hiroko Kudo et al. were able to detect Zn^2+^ in tap and river water with a 0.53 µM LOD by electrostatically immobilizing Zincon (2-carboxy-2′-hydroxy-5′-sulfoformazyl-benzene) onto a filter paper substrate in combination with highly water-absorbent materials [15]. Kathryn Chabaud et al. exploited the use of dithizone (1,5-diphenylthiocarbazone, DTZ) on Whatman No. 1 chromatography paper to detect Zn^2+^ (and other ions) in low explosives residue with a visual LOD of 25 ppm [16]. In a recent study, a colorimetric μPAD was developed to detect Zn^2+^ in urine using PAN (1-(2-pyridylazo)2-naphthol) on a chromatography paper (Grade 1 CHR) surface treated with 3-aminopropyltriethoxysilane and achieved a 35.9 μg L^−1^ LOD [3]. However, each study shows its own limitations. For example, authors have had to use different masking agents to minimize interference effects due to the presence of other ions [3,15,16]. A common limitation is in the fabrication process of these devices (for example, to create hydrophobic barriers), which is still intricate, is time-consuming, and sometimes requires specialized equipment. Manufacturing and limited stability are still two main factors that hamper the commercialization of μPADs and hamper their actual use in the real world [12].

In the present work, we describe the development of a qualitative colorimetric sensor for the detection of zinc ions in aqueous solutions. We used dithizone (1,5-diphenylthiocarbazone, DTZ) as zinc-chelating agent, exploiting the color change from yellow/orange to dark red/fuchsia with increasing zinc concentrations. Unlike traditional µPADs that almost exclusively use filter paper or chromatography paper [12], in this study, we evaluated different materials to develop a simple colorimetric device requiring fewer production steps. In addition, our goal was not to develop a device that improves the LOD of already published colorimetric μPADs but to develop a simple qualitative fit-for-purpose device that can reach the market more easily. The developed colorimetric sensor has been successfully applied to detect zinc ions in drinking water and urine, without needing sample preparation.

## 2. Materials and Methods

### 2.1. Chemicals

DTZ, pure ethanol, sodium carbonate, sodium bicarbonate, zinc sulphate heptahydrate, copper (II)-sulphate-pentahydrate, magnesium sulphate heptahydrate, calcium sulphate dihydrate, potassium peroxodisulphate, iron (II) sulphate heptahydrate, sodium sulphate, an iron standard for AAS, a lead standard for AAS, a cadmium standard for AAS and a mercury standard for AAS were purchased from Merck (Milan, Italy). Nickel (II) sulphate heptahydrate was purchased from Carlo Erba Reagents (Val de Reuil, France). All the chemicals were of analytical grade. All the solutions were prepared using ultrapurified water (18 MΩ·cm resistance, purified by a Purelab Prima system from Elga (Marlow, UK)).

### 2.2. Quantification of Urinary Zinc

Urinary zinc measurements were performed as previously reported [4]. Very briefly, samples were first acidified with 100 μL 18% *v*/*v* HCl and then diluted 1:5 with CsLaCl 1 g/L (1 mL sample + 4 mL CsLaCl 1 g/L). Once prepared, the samples were analyzed on the Zeenit 700p instrument (flame atomic absorption spectrometer, Analytik Jena). Four calibration curve standards were used for the analysis (S1: 4.5 mL CsLaCl 1 g/L + 400 μL H_2_O + 100 μL Std Cu/Zn 1 mg/L; S2: 4.5 mL CsLaCl 1 g/L + 500 μL Std Cu/Zn 0.5 mg/L; S3: 4.5 mL CsLaCl 1 g/L + 500 μL Std Cu/Zn 1 mg/L; S4: 4.5 mL CsLaCl 1 g/L + 500 μL Std Cu/Zn 2 mg/L) as well as two controls (Seronorm Urine L-1 and Seronorm Urine L-2) diluted 1:4 with CsLaCl 1 g/L (1 mL control + 3 mL CsLaCl 1 g/L). CsLaCl refers to cesium chloride–lanthanum chloride buffer solution and was used as an ionization suppressor in the flame.

### 2.3. Quantification of Zinc in Water Samples

Zinc measurements in ultrapure water, drinking water and Standard Reference Material^®^ NIST 1643f—trace element in water (Merck, Milan, Italy) (intended for the determination of trace elements in fresh water) were performed by GF-AAS (AAnalyst 600, Perkin Elmer, Norwalk, CT, USA). The quantification was performed at 213.9 nm using monoelemental Electrodeless Discharge Lamps. A transversely heated graphite atomizer with an integrated platform was used for the atomization of the samples, and the Zeeman effect was employed for background correction. Then, 20 μL of water sample was introduced into the furnace by an autosampler with a solution of Pd and Mg nitrate used as a matrix modifier. The heating program included a drying phase at 125 °C for 30 s, an ashing phase at 400 °C for 30 s, and an atomizing phase at 2500 °C for 10 s. An external standard calibration curve was used for Zn quantification in samples using metal ion solution prepared by dilution of commercial 1000 mg/L Zn stock solution (Merck, Milan, Italy). The analysis of ultrapure water was employed for the evaluation of the limit of detection (LOD) for the used AAS method (3.5 μg/L), while the measurement of SRM NIST 1643f was conducted to check its accuracy (obtained recovery 98 ± 3%).

### 2.4. Standard Solution Preparation for the Colorimetric Test

For the DTZ stock solution preparation, 10 mg of DTZ was dissolved in 10 mL of pure ethanol, resulting in a concentration of 1 mg/mL (3.95 mM). The solubilization involved vigorous stirring and sonication for 10 min. Subsequently, the prepared solution was diluted in a 0.5 M pH 9.6 carbonate buffer to achieve the desired concentration. This alkaline pH was chosen because in this condition, the DTZ-Zn^2+^ interaction is favored [17,18,19], even though it is reported that it can also happen at acidic pH [20]. In alkaline aqueous media, the thiol form of dithizone is favored and its solubility increases, and some interferences of other metal ions are reduced [21,22,23].

For the Zn^2+^ standard solution preparation, 14.4 mg of ZnSO_4_·7H_2_O was dissolved in 10 mL of ultrapure water, yielding a concentration of 1.44 mg/mL Zn^2+^ (5 mM). Other standard solutions of Zn^2+^ were then derived from the 5 mM solution through dilution with ultrapure water (or urine with Zn^2+^ concentration ca. 0.76 μΜ). The ultrapure water used for the experimental setup was analyzed by atomic absorption spectroscopy to ensure a near-zero concentration of Zn^2+^.

### 2.5. Colorimetric Sensor Development and Analytical Procedure

The procedure to obtain the colorimetric sensor is reported in Figure S1. Approximately 10 mL of DTZ diluted in carbonate buffer was poured into a 12 cm diameter Petri dish. An 81 cm^2^ square of the porous material was then immersed into the solution until saturated. The Petri dish was subjected to mild agitation on a shaker for a couple of minutes, and then the soaked material was transferred onto a silicone sheet and placed in an oven for 60 min at 40 °C. Subsequently, the 81 cm^2^ square of porous material was cut into smaller squares with dimensions of 0.25 cm^2^ using a CM4000 guillotine (Biodot, Irvine, CA, USA). The resulting squares were stored in plastic bags at room temperature until ready for use. The colorimetric squares can be used as they are or by placing them on a plastic support for easy handling.

To perform the analysis, a certain volume of the Zn^2+^ standard solution was dispensed onto the modified porous material. When not otherwise specified, the analyses were repeated in triplicate. Urinary samples and drinking water were analyzed without any pre-treatment. The colorimetric result was assessed after 10 min. For data reporting, images were acquired using an iPhone 13 (Apple Inc., Cupertino, CA, USA).

### 2.6. Materials for DTZ Deposition and Selection Criteria

The tested porous materials to be used for the DTZ deposition are listed in Table 1. Since we wanted to develop a qualitative colorimetric sensor, the selection criteria for selecting the most promising materials for the DTZ immobilization were based mainly on properties such as (i) color homogeneity of the material after drying, (ii) controlled sample diffusion (at least 5 mm from the sample deposition point) and (iii) uniform coloring detection (the material must avoid the washing effect that creates color gradients in the colorimetric measurements). Materials that show these characteristics allow for minimization of the production steps and therefore increase the chances of actual industrialization and commercialization of the device.

### 2.7. Fine-Tuning of the Colorimetric Sensor

During colorimetric sensor development, we assessed different aspects. Firstly, we evaluated the DTZ concentration in carbonate buffer to be deposited on the porous material. Three DTZ concentrations were evaluated, namely 100, 300 and 500 µg/mL. This preliminary evaluation was conducted using a cellulose-based material that is one of the most used for the development of paper-based colorimetric devices [14]. The DTZ concentration selection criterion was based on obtaining the best visual discrimination between different zinc concentrations (0, 50 and 500 µM). After that, other materials characterized by different porosity, thickness, and composition were assessed (Table 1). In addition to the aforementioned selection criteria for material selection, the principle for choosing the material to be used in the development of the sensor was to obtain a clear color change for the zinc concentrations of interest (at ca. 50 µM in drinking water [2] and ca. 25 µM in urine [4]).

In investigating the device’s sensitivity modulation (intended as the Zn concentration that causes a color change in the range of interest), different sample volumes (5, 10, 20, 30 µL) and different readout times (from 5 to 30 min) were also evaluated. The best sample volume must allow complete coverage of the porous material while providing homogeneous color development as well. The best readout time must ensure complete color development that does not change within a reading interval of at least 5 min, to allow sufficient time for result interpretation, minimizing possible user errors in complying with the recommended reading time.

The fine-tuning of the colorimetric device has been performed by analyzing fortified ultrapure water (ultrapure water was collected in the Chemistry Department).

### 2.8. Analytical Performance of the Colorimetric Sensor

Being designed as a qualitative test, to be used without a reader by a single final user, the output of the proposed device was evaluated visually by five different operators (to take into account variability due to subjective interpretation of the results) and their evaluations were used to assess sensor performance in terms of false negative and false positive rates, sensitivity, specificity and efficiency, where the false negative rate is the number of known negative samples that read positive; the false positive rate is the number of known positive samples that read negative; the sensitivity is the number of truly positive tests divided by the sum of known positive samples; the specificity is the number of truly negative tests divided by the sum of known negative samples; and the efficiency is the number of results correctly classified divided by the total of tests done (Table 2) [24,25,26,27].

Operators were asked whether they could observe a difference in color between the Zn^2+^ concentrations and/or to provide a color assessment. The operators’ assessment was considered valid and reliable only if at least 4 out of 5 operators gave the same assessment.

Knowing that the unreliability region of qualitative tests (the region where the relative rates of false responses are unacceptably high) is usually very close to the cut-off level, we set as the device performance acceptability criteria a maximum 15% false positive rate and 5% false negative rate at 0.5 and 2× the cut-off level (25 and 100 µM for tap water and 12.5 and 50 µM for urine samples). The more stringent criterion used for the false negative rate is justified by the fact that we wanted to reduce as much as possible the probability of missing tap water samples with bad potability quality and urinary patient samples with high risk of prostate cancer. This is because usually, samples with negative results provided by qualitative/screening tests are not analyzed again with a confirmatory method, while this happens for positive results.

Moreover, for the urine samples, we calculated the sensor accuracy in comparison to the reference atomic absorption spectrometer as the number of agreeing results divided by the number of samples measured (multiplied by 100). Concordance between the two methods was estimated by Cohen’s K.

#### 2.8.1. Selectivity of the Colorimetric Sensor and Shelf Life

To assess the applicability of the colorimetric sensor for real-sample analysis, the potential interference from different ions commonly found in aqueous matrices has been evaluated. Standard solutions of Cu^2+^, Ni^2+^, Na^+^, Ca^2+^, K^+^, Mg^2+^, Fe^2+^, Fe^3+^, Mn^2+^, Hg^2+^, Pb^2+^ and Cd^2+^ were analyzed using 20 µL as the sample volume and 10 min as the readout time.

The shelf life of the colorimetric sensor was evaluated by storing the devices (cellulose or nylon with DTZ immobilized) in foil pouches both at room temperature and at 37 °C over a period of 2 months. On the same day as device production (day 0), and after 7, 15, 30 and 60 days, the needed number of colorimetric sensors was taken from the foil pouches and was used to analyze the samples.

#### 2.8.2. Real-Sample Analysis

The reliability of the developed sensor for zinc detection has been evaluated by analyzing drinking water and urine samples. The drinking water was collected in the Chemistry Department. The water sample was analyzed as it was and after being fortified at two levels (25 and 100 µM) due to the difficulty of finding water samples with Zn concentration > ca. 50 µM.

Seventeen urine samples from patients scheduled for prostate biopsy at the urology clinic, Città della Salute e della Scienza di Torino—Molinette Hospital (Turin, Italy), previously analyzed by AAS, were tested with the colorimetric sensor. Urine samples were collected as previously reported [4], and their use was approved by the Scientific Ethics Committee of A.O.U. Città della Salute e della Scienza di Torino, A.O. Mauriziano, A.S.L. TO1 (Prot. No. 0110644). For the urine samples, we were also able to obtain the sensitivity and specificity of the device considering the clinical outcomes of the prostate biopsy.

Before the analysis of patients’ urinary samples, we evaluated the possible matrix effect on the colorimetric output. We prepared Zn^2+^ standard solution by fortifying a urine sample containing a very low Zn^2+^ concentration of 50 μg/L (ca. 0.76 μΜ), quantified by AAS.

## 3. Results

The goal of this work was to develop a fit-for-purpose qualitative sensor for the detection of Zn^2+^ in aqueous matrices, without the need for an external reader. The colorimetric sensor exploits the reaction between DTZ and Zn^2+^ to form colored chelates [19,20,23,28].

We decided to use DTZ since it is a well-established reagent in analytical chemistry due to its ability and selectivity in forming colored complexes, which are useful for trace metal analysis [21,22,29,30,31]. Moreover, the reaction selectivity between DTZ and metal ions can be modulated by adjusting the pH [21,22], while other zinc ligands like Zincon and PAN suffer from severe interferences that require additional masking agents to be avoided or minimized [3,15,16,32]. It has also been reported that the zinc–dithizone complex is more stable than its other metal complex [20,33].

The color of the chelates changes with increasing Zn^2+^ concentration, and therefore, after a proper fine-tuning, it can be used to assess the zinc level in the matrices of interests at desired cut-off concentrations. In our system, the colorimetric sensor starts with a yellowish color, turns orange and finally becomes red/fuchsia. The mechanism of the chelation process between Zn^2+^ and dithizone is reported in Figure S2.

### 3.1. Colorimetric Sensor Development and Fine-Tuning

To obtain a colorimetric sensor that can be used directly in situ, we decided to use porous adsorbent materials onto which the chromogenic reagent is deposited and stored in dried form. In the development process, it was important to evaluate the DTZ concentration to be used due to its relevant influence on the system’s sensitivity. In the starting experimental setup, a reference cellulose material (Whatman Grade 1) was employed to assess three different concentrations (ranging from 100 to 500 µg/mL) of DTZ diluted in carbonate buffer (50 mM, pH 9.6).

**Figure 1:**
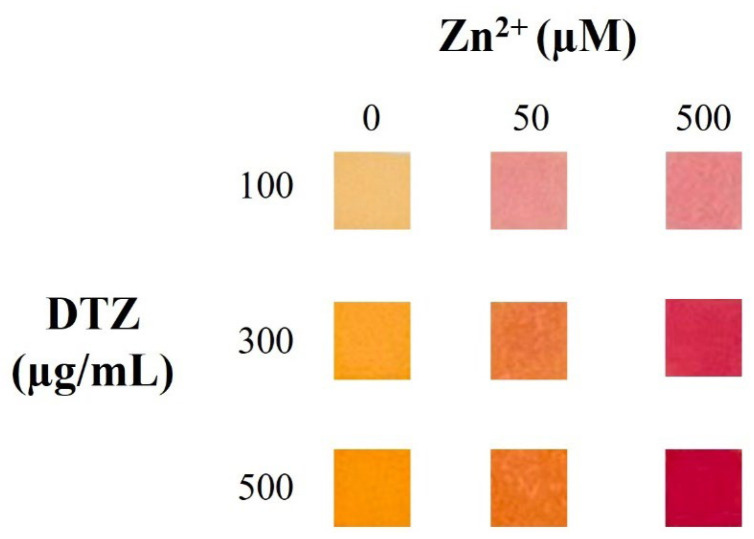
Effect of the DTZ concentration (100, 300 and 500 µg/mL), obtained by analyzing 10 μL of 0, 50 and 500 µM Zn^2+^ standard solutions prepared in ultrapure water.

The prepared colorimetric sensors were tested by analyzing 10 μL of 0, 50, and 500 µM Zn^2+^ standard solutions prepared in ultrapure water. The ultrapure water used in this work had a Zn^2+^ concentration below 3.5 µg/L (<53.5 nM), quantified by AAS. As reported in Figure 1, when using 100 µg/mL DTZ, discerning the color change between 50 and 500 µM Zn^2+^ concentrations was not possible: five out of five operators were unable to see any color change between 50 and 500 µM Zn^2+^, while a clear difference was appreciated by all of them between 0 and 50 µM Zn^2+^. In contrast, at 500 µg/mL DTZ, a clear color difference was observed between 50 and 500 µM Zn^2+^ (five out of five), while it was challenging to discriminate between 0 and 50 µM Zn^2+^ (one out of five). At 300 µg/mL DTZ, three distinct and easily distinguishable colors were observed, yielding enhanced resolution in terms of colorimetric discrimination. Therefore, we chose 300 µg/mL DTZ for further fine-tuning based on reduced reagent use while ensuring the best discrimination of various Zn^2+^ concentrations through observable color changes. The result interpretation provided by the five operators is reported in Table 3.

Operators were also asked to provide their interpretation by associating a color with the visual output observed (Table S1). This interpretation can be very useful in evaluating the performance of the device and further improving the use of the device if one is interested in obtaining semiquantitative results.

**Figure 2:**
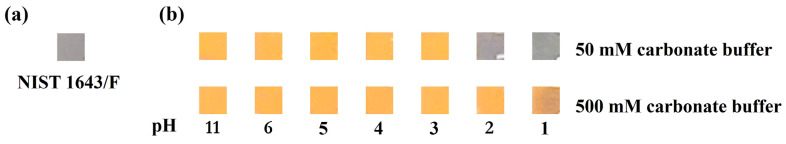
(**a**) Colorimetric output testing of 10 µL of certified water (NIST 1643/F). (**b**) Evaluation of pH interference using the colorimetric sensors obtained by preparing 300 µg/mL DTZ in 50 mM and 500 mM carbonate buffer.

To verify the output of the colorimetric sensor, we also analyzed an NST SRM^®^ solution that had a certified Zn^2+^ concentration of 74.4 ± 1.7 μg/L, ca. 1.1 µM (72.78 ± 0.26 μg/L measured by AAS). Based on the results reported in Figure 1, we should expect a dark-yellow output for the analysis of the certified Zn solution. On the contrary, as reported in Figure 2a, we obtained a gray colorimetric output that indicated an interference, probably due to the strongly acidic pH of the solution (pH ca. 0.6). The pH interference was further evaluated by using ultrapure water at different pH (from 1 to 6 and 11). Acidic interference was confirmed up to pH 2 (Figure 2b). Usually, drinking water samples and human urine samples do not show strongly acidic pH. However, to obtain a more versatile colorimetric sensor that could also be used to analyze highly acidic solutions, we tried to minimize this interference. To mitigate the acidic interference, we increased the concentration of the carbonate buffer used to prepare the 300 µg/mL DTZ solution from the DTZ stock solution. Increasing the carbonate concentration from 50 to 500 mM successfully mitigated the interference up to pH 1 (Figure 2b).

#### 3.1.1. Porous Material Evaluation and Selection

Different porous materials were saturated with 300 µg/mL DTZ prepared in 500 mM carbonate buffer at pH 9.6. In the process of material selection, we considered the color homogeneity of the material after drying in the preparation process and especially after sample deposition. The ideal material should guarantee the best color development homogeneity to avoid misleading result interpretation. In this phase, to evaluate whether the material itself may also contribute to the visual color thresholds (intended as the Zn^2+^ concentrations at which the operator is able to observe a color change), several Zn^2+^ standard solutions (0, 12.5, 25, 50, 100 and 500 µM) prepared in ultrapure water were analyzed (20 μL) and the colorimetric output was evaluated after 10 min (Figure S3).

Some of tested materials, such as those from SAATI and the polyethersulfone-based material from Cobetter, did not allow sufficient and homogeneous absorption of the sample, and they did not show a 5 mm diffusion from the sample deposition point. Whatman Std14 and Std17, and Alhstrom 1667 also did not show uniform coloring detection. Some materials (Alhstrom 1660 and Cobetter PSM180) did not show any color change from 0 to 500 µM Zn^2+^, while others lacked clear discrimination between zinc concentrations. Considering the intended use of the colorimetric sensor to analyze drinking water and urine samples, we selected those materials that allowed us to obtain a color change around the zinc concentration of interest (ca. 50 µM in drinking water [2] and ca. 25 µM in urine [4]). Therefore, Whatman Grade 1 and PALL Biodyne A were selected for further fine-tuning. The better performance shown by these materials can be attributed to their higher hydrophilicity that allows higher wettability. The color attribution provided by the operators is reported in Table S2. Based on their interpretation, it was possible to observe an orange–red transition between 25 and 50 µM Zn^2+^ using PALL Biodyne A and between 50 and 100 µM Zn^2+^ using Whatman Grade 1. These results showed that the nylon-based material provided an orange-to-red color change for lower Zn^2+^ concentrations in comparison to the cellulose-based material, suggesting that different materials can modulate the sensitivity of the colorimetric sensor. These differences may be due to the different absorption capacities of the materials. Therefore, different materials can be used for different goals, depending on the Zn^2+^ concentrations we want to discriminate. Specifically, PALL Biodyne A will be used for urine analysis (in which the cut-off is at a lower Zn^2+^ concentration), while Whatman Grade 1 will be used for drinking water analysis (in which the cut-off is at a higher Zn^2+^ concentration).

#### 3.1.2. Sample Volume and Readout Time

Sample volume and readout time may affect the device’s colorimetric output and visual color thresholds and were therefore evaluated for both the materials. Different sample volumes may result in different color output due to the different surface area of the DTZ-modified porous material that the sample can wet and interact with. Furthermore, small-volume samples may not diffuse 5 mm from the point of deposition, while larger-volume samples may not be absorbed effectively and may increase the likelihood of a washing effect that creates color gradients.

**Figure 3:**
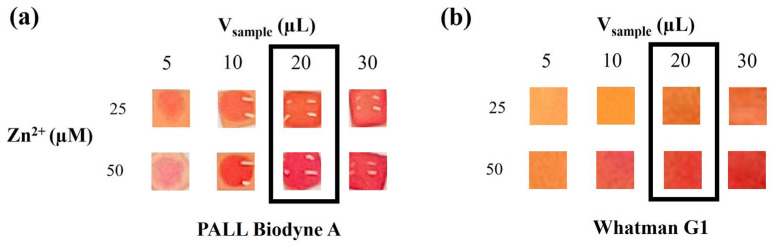
Assessment of color variation using different sample volumes (5, 10, 20, 30 µL) of Zn^2+^ standard solutions (25 and 50 µM) prepared in ultrapure water and using PALL Biodyne A (**a**) and Whatman Grade 1 (**b**) as porous material. The black rectangular line indicates the sample volume selected.

For the sample volume and readout time assessment, we decided to analyze the two cut-offs of interest (25 and 50 µM Zn^2+^). For Biodyne A, 5 and 10 µL were not enough to wet the whole colorimetric square (Figure 3a). Moreover, increasing the sample volume (from 20 to 30 µL) did not yield any sensitivity modulation. On the contrary, the highest volume tested (30 µL) showed a sort of color gradient, with the edge of the square characterized by a higher color intensity. Therefore, we chose 20 µL as the sample volume to be analyzed, since this volume ensured complete wettability and a homogeneous color development without color gradients. For Whatman Grade 1, even the smallest volume tested (5 µL) was able to wet the whole colorimetric square (Figure 3b). However, increasing the sample volume resulted in an increased color intensity output. The color intensity did not significantly increase from 20 to 30 µL. For this reason, for Whatman Grade 1, we also chose 20 µL as the best volume to be used in the analysis.

**Figure 4:**
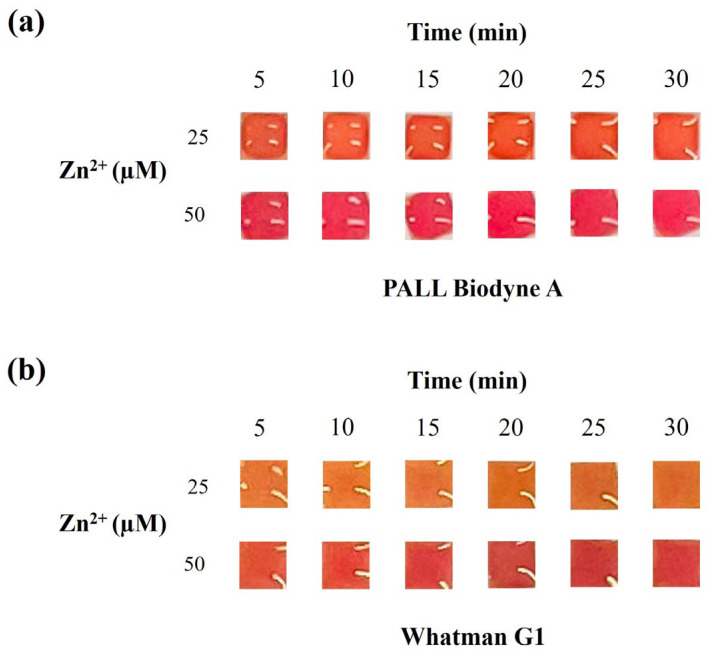
Assessment of color variation at different readout times (5, 10, 15, 20, 25 and 30 min) by analyzing Zn^2+^ standard solutions (25 and 50 µM) prepared in ultrapure water and using PALL Biodyne A (**a**) and Whatman Grade 1 (**b**) as porous material.

We also evaluated the readout time effect from 5 to 30 min on the color output. For both the materials, the colorimetric output did not change significantly over time (Figure 4); therefore, the results can be interpreted within 30 min. We suggested a readout time between 10 and 15 min. This wide readout time window is particularly advantageous in scenarios where the test is performed by unskilled personnel, as it ensures greater reliability and consistency in result interpretation.

#### 3.1.3. Selectivity

**Figure 5:**
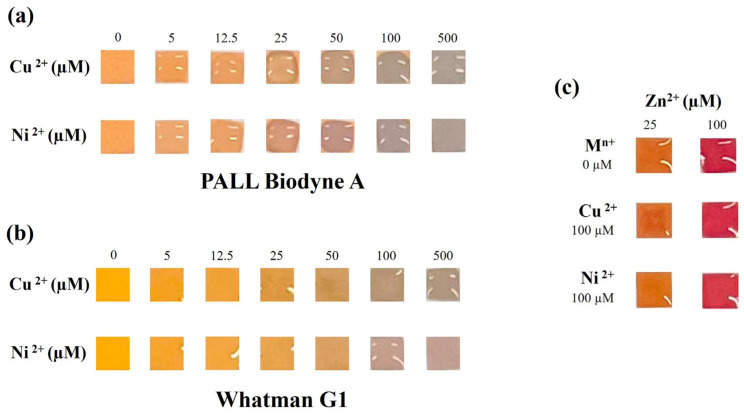
Interference threshold assessment by analyzing 20 μL Cu^2+^ and Ni^2+^ standard solutions (0, 5, 12.5, 25, 50, 100, 500 µM) prepared in ultrapure water and using PALL Biodyne A (**a**) and Whatman Grade 1 (**b**) as porous material. (**c**) Colorimetric output of binary mixtures Zn^2+^/Cu^2+^ (25 µM/100 µM and 100 µM/100 µM) and Zn^2+^/Ni^2+^ (25 µM/100 µM and 100 µM/100 µM) using Whatman Grade 1.

Before proving the applicability of the colorimetric sensor to real-sample analysis, the potential interference from other metallic cations commonly found in water and urine was assessed. In water, the most representative cations include calcium, magnesium, iron, manganese, copper, and sodium [34] followed by nickel, which is usually found in lower concentration. Of course, the occurrence of these cations may vary depending on environmental factors and sample origins. We analyzed standard solutions of the aforementioned cations and also of the heavy metals Cd^2+^, Pb^2+^ and Hg^2+^ at 100, 500 and 5000 µM. Na^+^, Ca^2+^, K^+^, Mg^2+^, Fe^2+^, Fe^3+^ and Mn^2+^ did not show any interference even at high concentration (Figure S4, Table S3), while Cu^2+^ and Ni^2+^ showed a clear interference (Figure 5). Therefore, for Cu^2+^ and Ni^2+^, we tested different concentrations to assess the maximum interference threshold.

Specifically, for PALL Biodyne A, Cu^2+^ and Ni^2+^ displayed interference starting from ca. 25 µM (Figure 5a, Table S4). For Whatman Grade 1, Cu^2+^ displayed interference starting from ca. 25 µM, while Ni^2+^ displayed interference at ca. 50 µM (Figure 5b, Table S4). Notably, interference from Cu^2+^ and Ni^2+^ manifested a unique gray-greenish color change. The legal limits for copper and nickel in drinking water are 2 mg/L (ca. 30 µM) and 20 µg/L (ca. 0.34 μM), respectively [35]. At these concentrations, Cu^2+^ and Ni^2+^ should not change the color output. However, acknowledging the evident interference from Cu^2+^ and Ni^2+^, we tested samples containing binary mixtures of zinc/copper and zinc/nickel to understand whether the interferent’s presence may affect correct zinc detection. We tested two Zn^2+^ concentrations, one clearly below the cut-off to prevent astringent taste in drinking water (0.5× cut-off: 25 µM) and one clearly above it (2× cut-off: 100 µM). Both Cu^2+^ and Ni^2+^ were tested at a concentration clearly above legal limits and known to cause a clear interference when analyzed alone (100 µM). Even for a high concentration of interferent ions, the colorimetric output for zinc detection has not been negatively affected (Figure 5c, Tables S5 and S6). These results demonstrate that the device can be used for accurate qualitative determination of Zn^2+^ even in the presence of interfering species at high concentrations. The absence of an interference effect may be due to a more favorable interaction between DTZ and Zn^2+^ than between DTZ and other metals when they are present simultaneously. At the same time, the device should guarantee accurate determination of Zn^2+^ in urine since the Ni^2+^ and Cu^2+^ urinary levels reported in the literature are much lower than the concentrations assessed (from 0.01 µM to 0.16 µM and from 0.2 µM to 8.5 µM, for Ni^2+^ and Cu^2+^, respectively [36,37,38,39,40]).

In addition, Hg^2+^ also showed clear interference (Figure S6, Table S7) starting from ca. 150 µM and ca. 250 µM for PALL Biodyne A and Whatman Grade 1, respectively. The legal limit for mercury in drinking water is 1 µg/L (ca. 5 nM) [35]. At this concentration, Hg^2+^ should not change the color output. At the same time, the device should guarantee accurate determination of Zn^2+^ in urine since the Hg^2+^ urinary levels reported in the literature are much lower than the concentrations that yield interference (from 0.5 nM to 25.6 nM [41,42,43,44], even for workers exposed to mercury (1.3 µM) [45]).

#### 3.1.4. Shelf Life

We evaluated the real-time and accelerated shelf life of the developed colorimetric sensor by analyzing two Zn^2+^ concentrations (0.5 and 2× cut-off) over a period of 2 months. This evaluation allowed us to check whether the colorimetric output for a certain Zn^2+^ concentration may change due to the aging of the device and whether the two selected Zn^2+^ concentrations were still distinguishable.

As shown in Figure S7, no significant color changes were observed over time, whether using the nylon- or cellulose-based porous material. The five operators were able to distinguish Zn^2+^ concentrations above and below the cut-off of interest with 100% efficiency, assigning the expected colors (Tables S8 and S9).

Therefore, the colorimetric sensor can be used up to 2 months from its production to obtain reliable results. Moreover, the accelerated stability evaluated at 37 °C suggests that the sensor’s shelf life can be even longer.

### 3.2. Water Analysis

Before the analysis of the tap water sample, the accuracy of the colorimetric sensor was further evaluated by analyzing ultrapure water fortified at 25 and 100 µM Zn^2+^ (0.5 and 2× cut-off) in ten replicates and considering the false positive and false negative rates. Based on the colorimetric threshold (Figure S3 and Table S2), a water sample was considered positive ([Zn^2+^] > cut-off) if the colorimetric output was red/fuchsia. On the contrary, a water sample was considered negative ([Zn^2+^] < cut-off) if the colorimetric output was (dark) yellow/orange.

**Figure 6:**
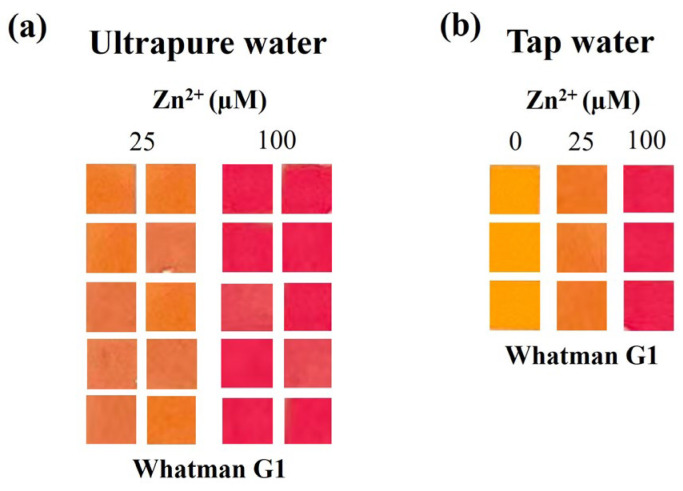
(**a**) Colorimetric output testing of 20 µL of ultrapure water fortified at 25 and 100 µM in 10 replicates; (**b**) colorimetric output testing of 20 µL of tap water fortified at 0, 25 and 100 µM in 3 replicates.

The Zn^2+^ content in the ultrapure water used has been quantified by AAS, with the result being <3.5 µg/L (<53.5 nM). The analyses demonstrated excellent repeatability (the operators always gave the same colorimetric assessment for the ten replicates), 0% false negative and 0% false positive rates, 100% sensitivity, 100% specificity and 100% efficiency (Figure 6a, Table S10).

Then, a sample of tap water was analyzed as it was and after being fortified at 25 and 100 µM Zn^2+^. The tap water as it was resulted in a yellowish color that is coherent with the Zn^2+^ content quantified by AAS (0.490 ± 0.004 mg/L, ca. 7.5 μM). Moreover, the tap water fortified at 25 and 100 µM Zn^2+^ resulted in the expected color changes, and again, 0% false negative and 0% false positive rates, 100% sensitivity, 100% specificity and 100% efficiency were obtained (Figure 6b, Table S11), showing that the developed sensor can be used for the reliable qualitative analysis of Zn^2+^ in drinking water.

### 3.3. Urine Analysis

Before the analysis of patients’ urinary samples, we evaluated the possible matrix effect on the colorimetric output. We prepared Zn^2+^ standard solutions by fortifying a urine sample containing a very low Zn^2+^ concentration of 50 μg/L (ca. 0.76 μΜ), quantified by AAS. We observed no significant matrix effect, apart from slightly less brilliant colors in comparison to water analysis (Figure S8). However, the color scale was as expected (Table S12). We again evaluated the readout time effect on the colorimetric output, and it was verified that a stable colorimetric output was reached within 5 min, and it was stable up to 30 min (Figure S9, Table S13). Moreover, the operators were always able to distinguish between 12.5 µM Zn^2+^ (0.5× cut-off) and 50 µM Zn^2+^ (2× cut-off) (Table S14). Therefore, for the urine analysis, we also suggest a readout time between 10 and 15 min.

**Figure 7:**
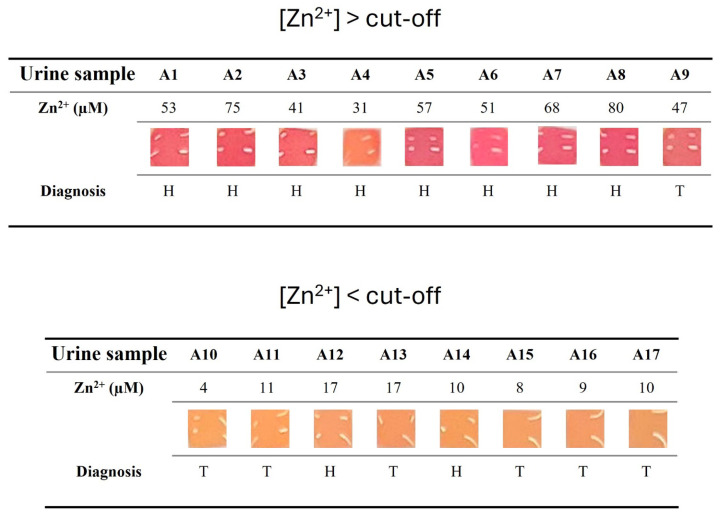
Colorimetric output testing of 20 µL of patients’ urinary samples using PALL Biodyne A as porous material. Zn^2+^ concentrations were obtained by AAS (H: healthy subject; T: patient with prostatic tumor).

Then, 17 samples previously analyzed through AAS were selected to assess the reliability of the colorimetric sensor in distinguishing samples with Zn^2+^ concentrations above or below the diagnostic cut-off value (ca. 25 µM) [5]. The patients’ urine samples were analyzed without any pre-treatment or dilution (Figure 7). Based on the colorimetric threshold (Figure S8 and Table S12), a urine sample was considered negative ([Zn^2+^] > cut-off) if the colorimetric output was red/fuchsia. On the contrary, a urine sample was considered positive ([Zn^2+^] < cut-off) if the colorimetric output was (dark) yellow/orange.

From the analytical point of view (considering whether the colorimetric output was coherent with the Zn^2+^ concentration quantified by AAS), the sensor showed an 11.1% false positive rate, 0% false negative rate, 100% sensitivity, 88.9% specificity and 94.1% efficiency (Figure 7, Table S15). Compared to the AAS, the sensor showed 94.1% accuracy and an almost-perfect concordance, expressed as Cohen’s kappa coefficient (κ = 0.88) [46]. This satisfying analytical performance was due to only one false positive result, sample A4, which, through AAS, gave a 32 µM Zn^2+^ concentration (to be considered negative, since it is above the 25 µM cut-off level), while with the colorimetric sensor, it gave an orange output (to be considered positive). However, this wrong classification is not surprising, since the Zn^2+^ was very close to the diagnostic cut-off where the probability of obtaining a false response is higher. Therefore, we can claim that the developed colorimetric sensor can be used for the reliable qualitative analysis of Zn^2+^ in urine samples.

Moreover, considering the classification of the urine samples based on their colorimetric output and the patients’ diagnosis after a prostate biopsy (H: healthy subject, which means that no tumor was found after the prostate biopsy; T: patient with prostatic tumor, which means that a tumor was found after the prostate biopsy), we obtained the diagnostic performance of the sensor for the small population analyzed. The sensor showed a 30% false positive rate, 14.3% false negative rate, 85.7% sensitivity, 70% specificity and 76.5% efficiency. This diagnostic performance was due to three false positive results (samples A4, A12, A14) and one false negative result (sample A9). The slightly worse diagnostic performance compared to the analytical one is due to the inherent error associated with the definition of the clinical cut-off.

### 3.4. Comparison with Commercially Available Test Strip

In addition to the colorimetric μPADs already mentioned for Zn^2+^ detection [3,17,18], some devices are also commercially available. Among them, the QUANTOFIX Zink by Macherey-Nagel (Düren, Germany) is the most well-known. It is worth noting that this test is not designed for Zn^2+^ determination in urine, and in general, none of the current urine test strips commercially available allows Zn^2+^ determination.

Based on the technical documentation shared by the manufacturer on the website [47], the QUANTOFIX Zink is less sensitive in comparison to the developed device (first point of colorimetric calibration: 2 mg/L Zn^2+^, ca. 30.6 μM). Moreover, it requires additional steps, an additional reagent, and a higher volume of sample (5 mL). Therefore, our system has the potential to be a more convenient and easier way to assess the presence of Zn^2+^ both in drinking water and in urine.

## 4. Conclusions

In this study, we developed an easy and straightforward colorimetric sensor for the visual detection of Zn^2+^ in aqueous matrices without any pre-treatment. The rapid colorimetric output is due to the color change of the complexant dithizone in the presence of Zn^2+^. Cellulose- and nylon-based materials were revealed to be the most suitable materials for the immobilization of the dithizone in dried form. In the evaluated setup, cellulose-based material should be preferred for drinking water analysis, while nylon-based material is more appropriate for urinary sample analysis (due to the different Zn^2+^ cut-off level). The manufacturing of the sensor is easy and does not need the formation of hydrophobic barriers that are commonly needed for traditional μPADs. The colorimetric sensor is selective and showed excellent concordance with the reference method.

Even if the sensor is intended for qualitative visual Zn^2+^ detection, the analysis can be further improved and result interpretation be made more objective by exploiting image analysis and by using a color scale as a reference. To this end, a digital color scale with unique HEX (RGB) codes identified using Microsoft Office software is provided in Figure S10. With this implementation, the sensor also has the potential to be used for Zn^2+^ semiquantitative analysis in aqueous matrices. Moreover, the optimization of the sensor should improve the analytical sensitivity and allow its use for those applications in which a lower concentration of Zn^2+^ needs to be detected.

## Figures and Tables

**Table 1:** List of materials investigated for the deposition of the colorimetric reagent for the determination of Zn^2+^ in aqueous solutions.

Supplier	Material Name	Material Composition
Whatman Cytiva (Maidstone, UK)	Grade CF1	100% cotton linter
	AE100	Cellulose nitrate
	Standard 14	Glass fiber
	Standard 17	
	Grade 1	Cellulose
	LF1	Bound glass fiber
Ahlstrom-Munksjö (Helsinki, Finland)	1667	Proprietary fiber blend
	1660	New patented fiber blend
PALL Corp (Port Washington, NY, USA)	Vivid PS	Asymmetric polysulfone
	Biodyne A	Amphoteric Nylon 6.6
SAATI (Appiano Gentile, Italy)	0.1 μm	Polyamide 6
	0.5 μm	
	1 μm	
Cobetter filtration (Hangzhou, China)	JN6 0.65	Nylon
	PSM180	Hydrophilic and highly asymmetric polyethersulfone
	PES IV 1.2 S	Polyethersulfone
	PES IV 0.2B	
	PES AEX 0.45	

**Table 2:** Performance characteristics for the evaluation of the qualitative colorimetric sensor.

False Negative Rate	False Positive Rate	Sensitivity	Specificity	Efficiency
FN/(TP + FN)	FP/(TN + FP)	TP/(TP + FN)	TN/(TN + FP)	(TP + TN)/(TP + FP + TN + FN)

FN: number of false negatives; TN: number of true negatives; FP: number of false positives; TP: number of true positives.

**Table 3:** Results for the analysis of fortified ultrapure water (10 µL of 0, 50 and 500 μM Zn^2+^) to study the effect of the DTZ concentration (100, 300 and 500 µg/mL). Qualitative evaluation was performed by 5 operators in three replicates.

		Zn^2+^ (µM)		
DTZ (µg/mL)		0 vs. 50	0 vs. 500	50 vs. 500
	100	+ + + + +	+ + + + +	− − − − −
	300	+ + + + +	+ + + + +	+ + + + +
	500	− − − + −	+ + + + +	+ + + + +

+: The operator was able to observe a color difference between the two Zn^2+^ concentrations. −: The operator was not able to observe a color difference between the two Zn^2+^ concentrations.

## Data Availability

The raw data supporting the conclusions of this article will be made available by the authors on request.

## Supplementary Materials

The following supporting information can be downloaded at https://www.mdpi.com/article/10.3390/s26061926/s1. Figure S1: Stepwise procedure to obtain the colorimetric sensor. Figure S2: The mechanism of the chelation process between Zn^2+^ and dithizone. In slightly alkaline aqueous media, the thiol form of dithizone is favored and interacts with Zn^2+^ in a 1:2 metal-to-ligand ratio. Figure S3: Appearance of different porous materials modified with DTZ 300 μg/mL prepared in 500 mM carbonate buffer after the deposition of 20 μL Zn^2+^ standard solutions (0, 12.5, 25, 50, 100 and 500 µM) prepared in ultrapure water. Figure S4: Interference assessment by analyzing 20 μL Na^+^, Ca^2+^, K^+^, Mg^2+^, Fe^2+^ and Mn^2+^ standard solutions (100, 500, 5000 µM) prepared in ultrapure water and using PALL Biodyne A (**a**) and Whatman Grade 1 (**b**) as porous material. Ref indicates the output of the analysis of 20 μL ultrapure water. Figure S5: Interference assessment by analyzing 20 μL Cd^2+^, Pb^2+^ and Fe^3+^ standard solutions (100, 500, 5000 µM) prepared in ultrapure water and using PALL Biodyne A (**a**) and Whatman Grade 1 (**b**) as porous material. Ref indicates the output of the analysis of 20 μL ultrapure water. The iPhone 13 was not available anymore in our lab, so the additional experiments requested during the peer-review were documented using an iPhone 16 Pro. Figure S6: Interference threshold assessment by analyzing 20 μL Hg^2+^ standard solutions (0, 5, 12.5, 25, 50, 100, 150, 250, 500 µM) prepared in ultrapure water and using PALL Biodyne A and Whatman Grade 1 as porous material. The iPhone 13 was not available anymore in our lab, so the additional experiments requested during the peer-review were documented using an iPhone 16 Pro. Figure S7: Real-time (25 °C) and accelerated (37 °C) shelf life for the colorimetric sensor at days 0, 7, 15, 30 and 60, obtained by analyzing 20 μL of Zn^2+^ standard solutions (0.5 and 2× cut-off): 12.5 and 50 µM using PALL Biodyne A (**a**) and 25 and 100 µM using Whatman Grade 1 (**b**) as porous material. Figure S8: Assessment of color variation by analyzing Zn^2+^ standard solutions (0, 12.5, 25, 50, 100 and 500 µM) prepared in urine sample (0.76 μΜ Zn^2+^) and using PALL Biodyne A as porous material. Figure S9: Assessment of color variation at different readout times (1, 5, 10 and 30 min) by analyzing Zn^2+^ standard solutions (12.5 and 50 µM) prepared in urine sample (0.76 μΜ Zn^2+^) and using PALL Biodyne A as porous material. Figure S10: Digital color scale with unique HEX (RGB) codes identified using Microsoft Office software for PALL Biodyne A and Whatman Grade 1. Table S1: Results for the analysis of fortified ultrapure water (10 µL of 0, 50 and 500 µM Zn^2+^) to study the effect of the DTZ concentration (100, 300 and 500 µg/mL). The color assignment was performed by 5 operators in three replicates. Table S2: Results for the analysis of fortified ultrapure water (20 µL of 0, 12.5, 25, 50, 100 and 500 µM Zn^2+^) using PALL Biodyne A and Whatman Grade 1 as porous material. Qualitative evaluation was performed by 5 operators in three replicates. Table S3: Interference threshold assessment by analyzing 20 μL Na^+^, Ca^2+^, K^+^, Mg^2+^, Fe^2+^, Fe^3+^, Mn^2+^, Cd^2+^ and Pb^2+^ standard solutions (100, 500 and 5000 µM) prepared in ultrapure water and using PALL Biodyne A and Whatman Grade 1 as porous material. The interference threshold was assessed by comparing the Mn^+^ standard solution output with a reference (Ref) output obtained by analyzing non-fortified ultrapure water. The comparison was performed by 5 operators. Table S4: Interference threshold assessment by analyzing 20 μL Cu^2+^ and Ni^2+^ standard solutions (5, 12.5, 25, 50, 100 and 500 µM) prepared in ultrapure water and using PALL Biodyne A and Whatman Grade 1 as porous material. The interference threshold was assessed by comparing the Mn+ standard solution output with the output obtained by analyzing non-fortified ultrapure water. The comparison was performed by 5 operators. Table S5: Interference assessment by analyzing 20 μL of binary mixtures Zn^2+^/Cu^2+^ (25 µM/100 µM and 100 µM/100 µM) and Zn^2+^/Ni^2+^ (25 µM/100 µM and 100 µM/100 µM) using Whatman Grade 1 as porous material. The interference effect was assessed by checking whether operators were still able to correctly distinguish between 25 and 100 µM Zn^2+^ in the presence of 100 µM of interfering species. The comparison was performed by 5 operators. Table S6: Interference assessment by analyzing 20 μL of binary mixtures Zn^2+^/Cu^2+^ (25 µM/100 µM and 100 µM/100 µM) and Zn^2+^/Ni^2+^ (25 µM/100 µM and 100 µM/100 µM) using Whatman Grade 1 as porous material. The interference effect was assessed by checking whether operators were able to observe a color difference between the Zn^2+^ standard solutions (25 and 100 µM) in the absence of and in the presence of 100 µM of interfering species. The comparison was performed by 5 operators. Table S7: Interference threshold assessment by analyzing 20 μL Hg^2+^ standard solutions (5, 12.5, 25, 50, 100, 150, 250 and 500 µM) prepared in ultrapure water and using PALL Biodyne A and Whatman Grade 1 as porous material. The interference threshold was assessed by comparing the M^n+^ standard solution output with the output obtained by analyzing non-fortified ultrapure water. The comparison was performed by 5 operators. Table S8: Shelf-life evaluation at 25 and 37 °C up to 2 months. The color assignment for the analysis of fortified ultrapure water (20 µL of 12.5 µM and 50 µM or 20 µL of 25 µM and 100 µM Zn^2+^) using PALL Biodyne A and Whatman Grade 1 as porous material was performed by 5 operators in three replicates. Table S9: Shelf-life evaluation at 25 and 37 °C up to 2 months. The ability of the device to distinguish Zn^2+^ concentrations above and below the cut-off (20 µL of 12.5 µM vs. 50 µM or 20 µL of 25 µM vs. 100 µM Zn^2+^, prepared in ultrapure water), using PALL Biodyne A and Whatman Grade 1 as porous material, was qualitatively evaluated by 5 operators in three replicates. Table S10: Results for the analysis of fortified ultrapure water (20 µL of 25 and 100 µM Zn^2+^) using Whatman Grade 1 as porous material. Qualitative evaluation was performed by 5 operators in ten replicates. Table S11: Results for the analysis of fortified tap water (20 µL of 0, 25 and 100 µM Zn^2+^) using Whatman Grade 1 as porous material. Qualitative evaluation was performed by 5 operators in three replicates. Table S12: Results for the analysis of fortified urine (20 µL of 0, 12.5, 25, 50, 100 and 500 µM Zn^2+^) using PALL Biodyne A as porous material. Qualitative evaluation was performed by 5 operators in three replicates. Table S13: Assessment of reading time effect (1, 5, 10 and 30 min) by analyzing Zn^2+^ standard solutions (20 µL of 12.5 and 50 μM) prepared in urine sample (0.76 μΜ Zn^2+^) and using PALL Biodyne A as porous material. Qualitative evaluation was performed by 5 operators in three replicates. Table S14: Assessment of color variation at different readout times (1, 5, 10 and 30 min) by analyzing Zn^2+^ standard solutions (20 µL of 12.5 and 50 μM) prepared in urine sample (0.76 μΜ Zn^2+^). The ability of the device to distinguish the two Zn^2+^ concentrations, using PALL Biodyne A as porous material, was qualitatively evaluated by 5 operators in three replicates. Table S15: Results for the analysis of patients’ urinary samples using PALL Biodyne A as porous material. Qualitative evaluation was performed by 5 operators in three replicates.
