# Supplementary material for: A Rapid and Versatile Colorimetric Sensor for the Visual Detection of Zinc Ions in Urine and Drinking Water
Source: Sensors (Basel). 2026 Mar 19;26(6):1926. doi: 10.3390/s26061926 (PMC13030703; doi:10.3390/s26061926)
Supplement: Supplementary file 1 [file sensors-26-01926-s001.zip › sensors-4171030-supplementary.pdf]

SUPPLEMENTARY MATERIAL

A rapid and versatile colorimetric sensor for the visual detection of zinc ions in urine and drinking water

Sensors

Thea Serra[1], Fabio Di Nardo[1]*, Simone Cavalera[1], Valentina Testa[1], Stefano Bertinetti[1], Claudio Baggiani[1], Daniele Amparore[2], Sabrina De Cillis[2], Sergio Occhipinti[3,4], Laura Anfossi[1]

[1]Department of Chemistry, University of Turin, 10125, Turin, Italy

[2]Department of Oncology, University of Turin, 10043, Orbassano, Italy

[3]Department of Molecular Biotechnologies and Health Sciences, University of Turin, 10126, Turin, Italy

[4]NIB biotec srl, Via Candido Viberti 4, 10141, Turin, Italy

* Corresponding author

Prof. Fabio Di Nardo, Ph.D.

Dipartimento di Chimica, Università di Torino. Via Giuria 7, Torino, Italy

Email: fabio.dinardo@unito.it

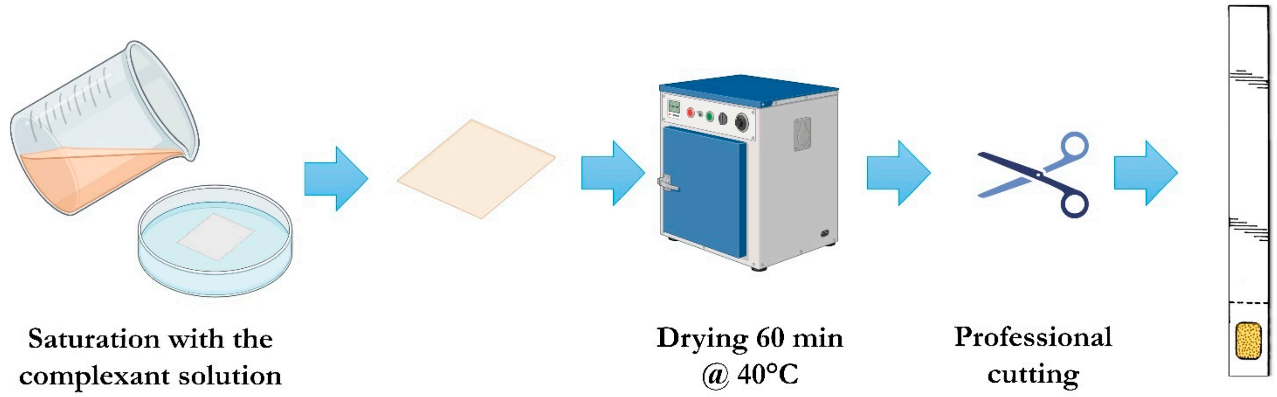

Figure S1. Stepwise procedure to obtain the colorimetric sensor.

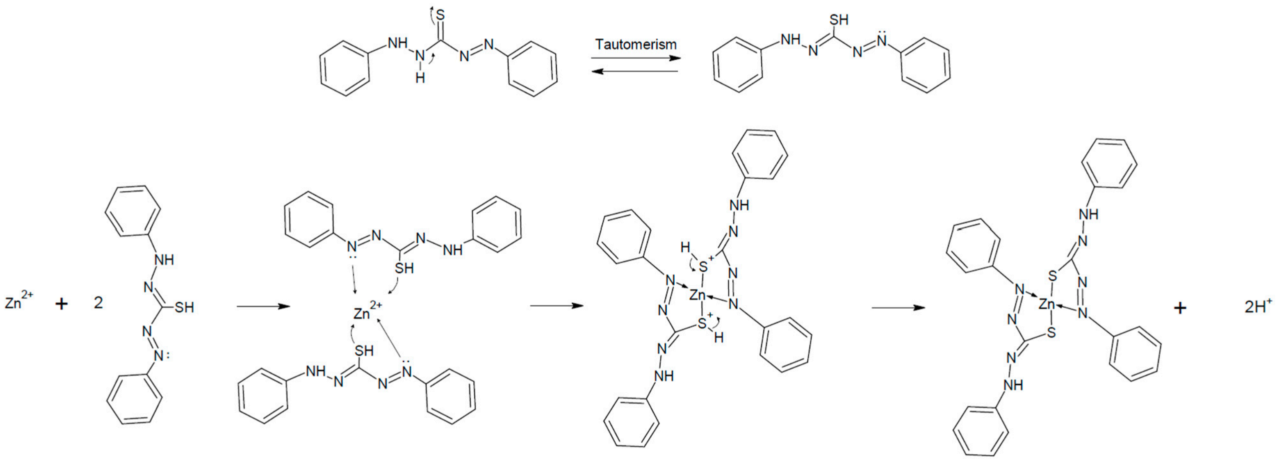

Figure S2. The mechanism of the chelation process between Zn^{2+} and dithizone. In slightly alkaline aqueous media, the thiol form of dithizone is favored and interacts with Zn^{2+} in a 1:2 metal-to-ligand ratio.

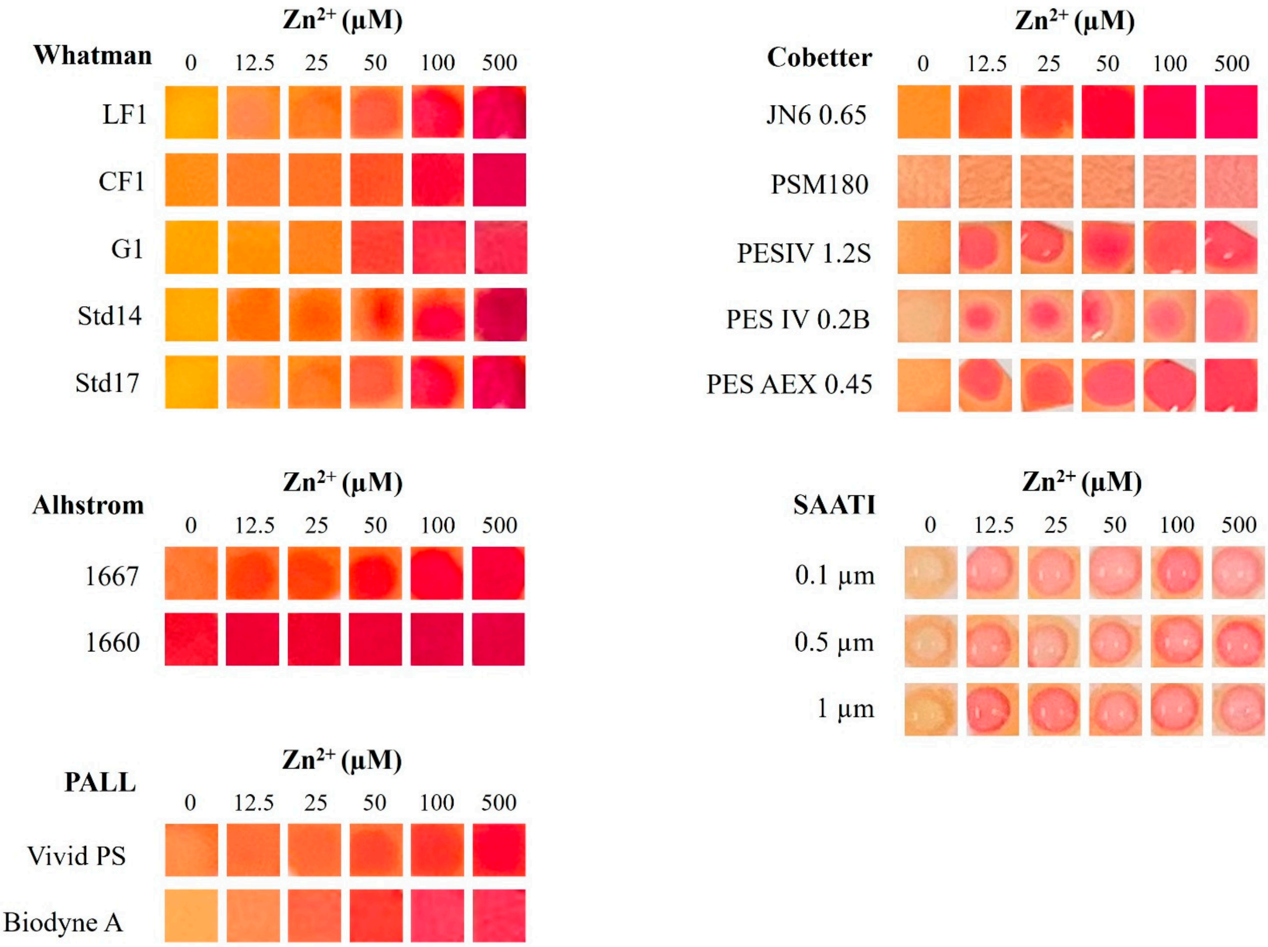

Figure S3. Appearance of different porous materials modified with DTZ 300 µg/mL prepared in 500 mM carbonate buffer after the deposition of 20 µL Zn^{2+} standard solutions (0, 12.5, 25, 50, 100 and 500 µM) prepared in ultrapure water.

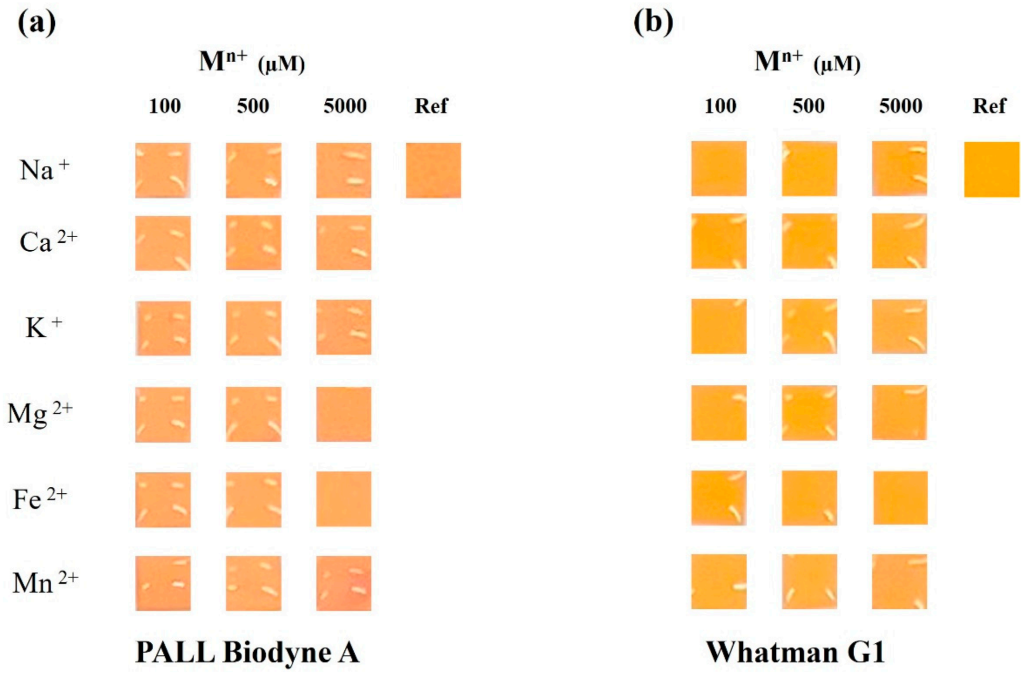

Figure S4. Interference assessment by analyzing 20 µL Na^{+}, Ca^{2+}, K^{+}, Mg^{2+}, Fe^{2+} and Mn^{2+} standard solutions (100, 500, 5000 µM) prepared in ultrapure water and using PALL Biodyne A (a) and Whatman Grade 1 (b) as porous material. Ref indicates the output of the analysis of 20 µL ultrapure water.

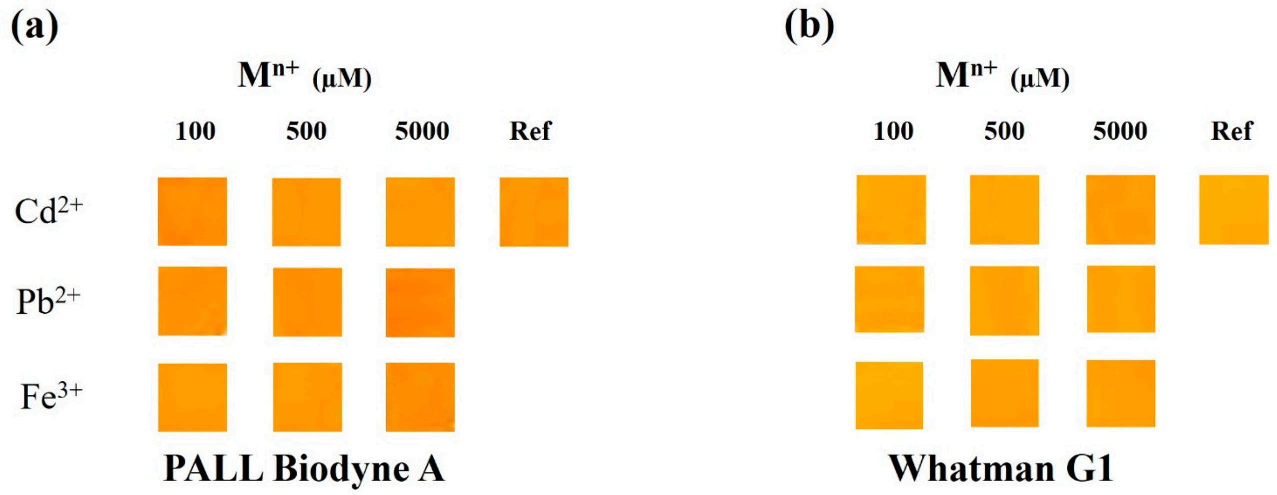

Figure S5. Interference assessment by analyzing 20 µL Cd^{2+}, Pb^{2+} and Fe^{3+} standard solutions (100, 500, 5000 µM) prepared in ultrapure water and using PALL Biodyne A (a) and Whatman Grade 1 (b) as porous material. Ref indicates the output of the analysis of 20 µL ultrapure water. The iPhone 13 was not available anymore in our lab, so the additional experiments requested during the peer-review were documented using an iPhone 16 Pro.

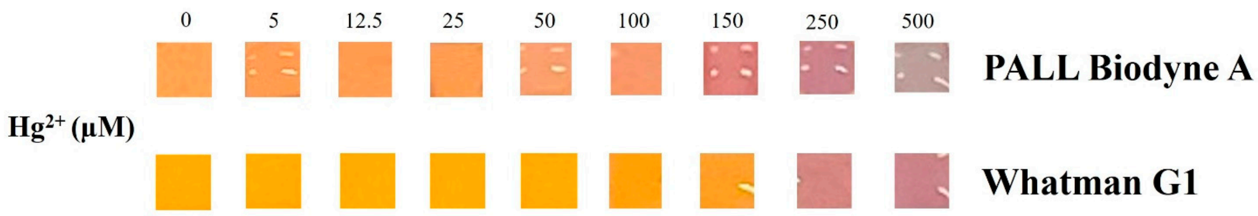

Figure S6. Interference threshold assessment by analyzing 20 µL Hg^{2+} standard solutions (0, 5, 12.5, 25, 50, 100, 150, 250, 500 µM) prepared in ultrapure water and using PALL Biodyne A and Whatman Grade 1 as porous material. The iPhone 13 was not available anymore in our lab, so the additional experiments requested during the peer-review were documented using an iPhone 16 Pro.

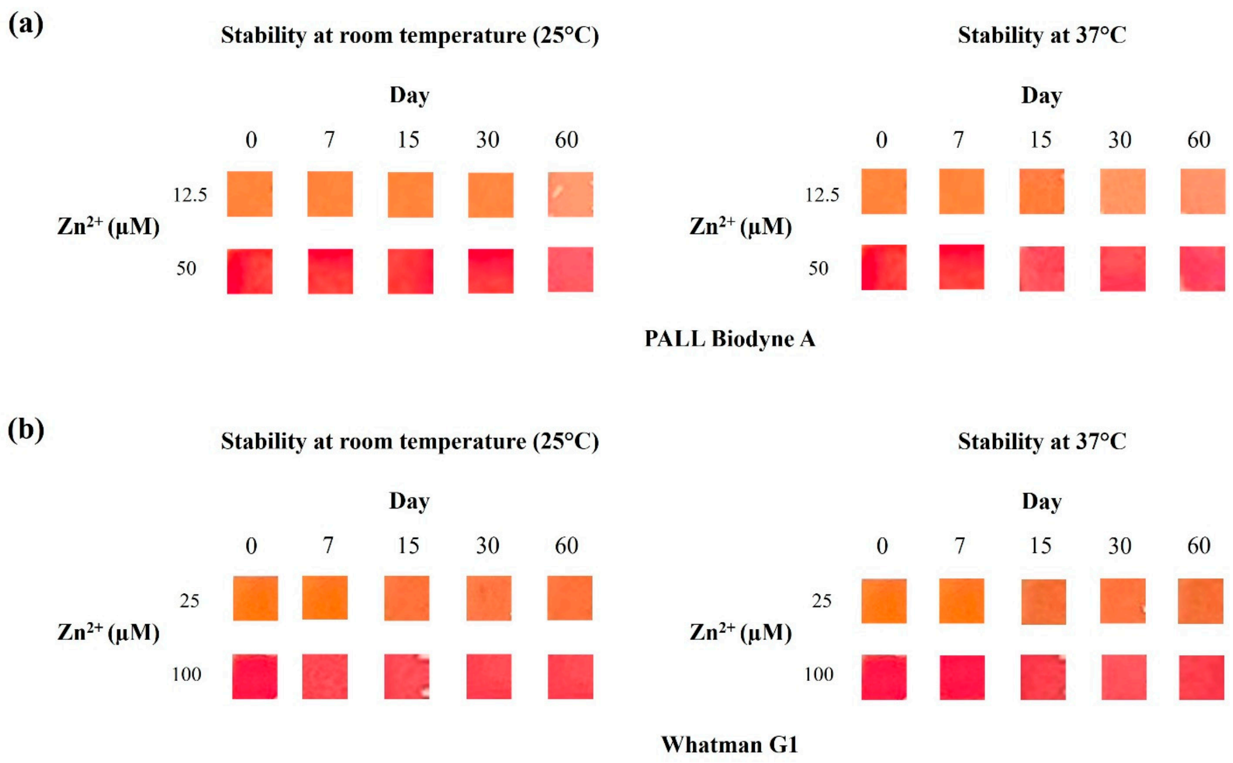

Figure S7. Real-time (25°C) and accelerated (37°C) shelf life for the colorimetric sensor at days 0, 7, 15, 30 and 60, obtained by analyzing 20 µL of Zn^{2+} standard solutions (0.5 and 2x cut-off): 12.5 and 50 µM using PALL Biodyne A (a) and 25 and 100 µM using Whatman Grade 1 (b) as porous material.

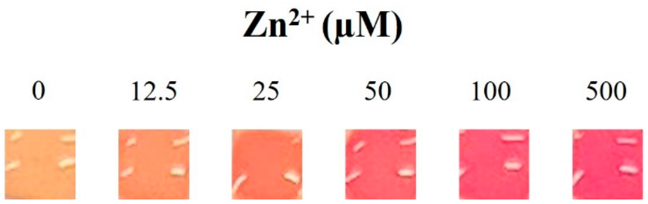

Figure S8. Assessment of color variation by analyzing Zn^{2+} standard solutions (0, 12.5, 25, 50, 100 and 500 µM) prepared in urine sample (0.76 µM Zn^{2+}) and using PALL Biodyne A as porous material.

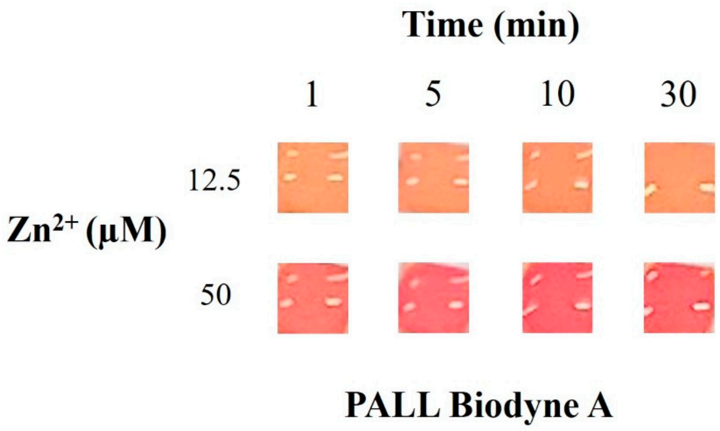

Figure S9. Assessment of color variation at different readout times (1, 5, 10 and 30 min) by analyzing Zn^{2+} standard solutions (12.5 and 50 µM) prepared in urine sample (0.76 µM Zn^{2+}) and using PALL Biodyne A as porous material.

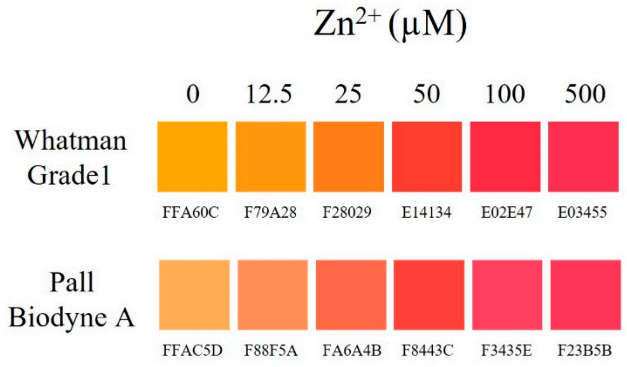

Figure S10. Digital color scale with unique HEX (RGB) codes identified using Microsoft Office software for PALL Biodyne A and Whatman Grade 1.

Table S1. Results for the analysis of fortified ultrapure water (10 µL of 0, 50 and 500 µM Zn^{2+}) to study the effect of the DTZ concentration (100, 300 and 500 µg/mL). The color assignment was performed by 5 operators in three replicates.

		Zn^{2+} (µM)		
		0	50	500
DTZ (µg/mL)	100	LY, LY, LY, LY, LY	LP, LP, LP, LP, LP	LP, LP, LP, LP, LP
	300	DY, DY, DY, DY, DY	DO, DO, DO, DO, DO	DR, DR, DR, DR, DR
	500	DY, O, O, O, O	DO, DO, DO, DO, DO	DR, DR, DR, DR, DR

LY: light yellow; LP: light pink; DY: dark yellow; DO: dark orange; DR: dark red; O: orange.

Table S2. Results for the analysis of fortified ultrapure water (20 µL of 0, 12.5, 25, 50, 100 and 500 µM Zn^{2+}) using PALL Biodyne A and Whatman Grade 1 as porous material. Qualitative evaluation was performed by 5 operators in three replicates.

	Zn^{2+} (µM)					
	0	12.5	25	50	100	500
**PALL Biodyne A**	Y, DY, DY, DY, DY	LO, LO, LO, LO, O	O, O, O, O, O	LR, R, R, LR, R	DR, F, DR, F, DR	DR, F, F, F, DR
**Whatman Grade 1**	DY, DY, DY, DY, DY	DY, LO, O, LO, LO	O, O, O, O, O	DO, DO, DO, DO, R	R, R, R, R, R	R, R, DR, R, DR

Y: yellow; DY: dark yellow; LO: light orange, O: orange; DO: dark orange; LR: light red; R: red; DR: dark red; F: fuchsia.

Table S3. Interference threshold assessment by analyzing 20 µL Na^+, Ca^{2+}, K^+, Mg^{2+}, Fe^{2+}, Fe^{3+}, Mn^{2+}, Cd^{2+} and Pb^{2+} standard solutions (100, 500 and 5000 µM) prepared in ultrapure water and using PALL Biodyne A and Whatman Grade 1 as porous material. The interference threshold was assessed by comparing the M^{n+} standard solution output with a reference (Ref) output obtained by analyzing non-fortified ultrapure water. The comparison was performed by 5 operators.

		M^{n+} (µM)								
	**Comparison**	Na^+	Ca^{2+}	K^+	Mg^{2+}	Fe^{2+}	Fe^{3+}	Mn^{2+}	Cd^{2+}	Pb^{2+}
**PALL Biodyne A**	Ref vs 100	−,−,−,−,−	−,−,−,−,−	−,−,−,−,−	−,−,−,−,−	−,−,−,−,−	−,−,−,−,−	−,−,−,−,−	−,−,−,−,−	−,−,−,−,−
	Ref vs 500	−,−,−,−,−	−,−,−,−,−	−,−,−,−,−	−,−,−,−,−	−,−,−,−,−	−,−,−,−,−	−,−,−,−,−	−,−,−,−,−	−,−,−,−,−
	Ref vs 5000	−,−,−,−,−	−,−,−,−,−	−,−,−,−,−	−,−,−,−,−	−,−,−,−,−	−,−,−,−,−	−,−,−,−,−	−,−,−,−,−	−,−,−,−,−
**Whatman Grade 1**	Ref vs 100	−,−,−,−,−	−,−,−,−,−	−,−,−,−,−	−,−,−,−,−	−,−,−,−,−	−,−,−,−,−	−,−,−,−,−	−,−,−,−,−	−,−,−,−,−
	Ref vs 500	−,−,−,−,−	−,−,−,−,−	−,−,−,−,−	−,−,−,−,−	−,−,−,−,−	−,−,−,−,−	−,−,−,−,−	−,−,−,−,−	−,−,−,−,−
	Ref vs 5000	−,−,−,−,−	−,−,−,−,−	−,−,−,−,−	−,−,−,−,−	−,−,−,−,−	−,−,−,−,−	−,−,−,−,−	−,−,−,−,−	−,−,−,−,−

−: The operator was not able to observe a color difference between the M^{n+} and the Ref output.

Table S4. Interference threshold assessment by analyzing 20 µL Cu^{2+} and Ni^{2+} standard solutions (5, 12.5, 25, 50, 100 and 500 µM) prepared in ultrapure water and using PALL Biodyne A and Whatman Grade 1 as porous material. The interference threshold was assessed by comparing the M^{n+} standard solution output with the output obtained by analyzing non-fortified ultrapure water. The comparison was performed by 5 operators.

		M^{n+} (µM)					
	**Comparison**	0 vs 5	0 vs 12.5	0 vs 25	0 vs 50	0 vs 100	0 vs 500
**PALL Biodyne A**	Cu^{2+}	−, −, −, −, −	−, −, −, −, −	+, +, +, +, +	+, +, +, +, +	+, +, +, +, +	+, +, +, +, +
	Ni^{2+}	−, −, −, −, −	−, −, −, −, −	+, +, +, +, +	+, +, +, +, +	+, +, +, +, +	+, +, +, +, +
**Whatman Grade 1**	Cu^{2+}	−, −, −, −, −	−, −, −, −, −	+, +, +, +, +	+, +, +, +, +	+, +, +, +, +	+, +, +, +, +
	Ni^{2+}	−, −, −, −, −	−, −, −, −, −	−, −, −, −, −	+, +, +, +, +	+, +, +, +, +	+, +, +, +, +

−: The operator was not able to observe a color difference between the two M^{n+} standard solutions.
+: The operator was able to observe a color difference between the two M^{n+} standard solutions.

Table S5. Interference assessment by analyzing 20 µL of binary mixtures Zn^{2+}/Cu^{2+} (25 µM/100 µM and 100 µM/100 µM) and Zn^{2+}/Ni^{2+} (25 µM/100 µM and 100 µM/100 µM) using Whatman Grade 1 as porous material. The interference effect was assessed by checking whether operators were still able to correctly distinguish between 25 and 100 µM Zn^{2+} in the presence of 100 µM of interfering species. The comparison was performed by 5 operators.

		**Zn^{2+} (µM)**
		25 vs 100
**M^{n+} (100 µM)**	Cu^{2+}	+, +, +, +, +
	Ni^{2+}	+, +, +, +, +

+: The operator was able to observe a color difference between the two Zn^{2+} standard solutions (25 and 100 µM) in the presence of 100 µM Cu^{2+} or Ni^{2+}.

Table S6. Interference assessment by analyzing 20 µL of binary mixtures Zn^{2+}/Cu^{2+} (25 µM/100 µM and 100 µM/100 µM) and Zn^{2+}/Ni^{2+} (25 µM/100 µM and 100 µM/100 µM) using Whatman Grade 1 as porous material. The interference effect was assessed by checking whether operators were able to observe a color difference between the Zn^{2+} standard solutions (25 and 100 µM) in the absence of and in the presence of 100 µM of interfering species. The comparison was performed by 5 operators.

**Comparison**			
25 µM Zn^{2+} Vs 100 µM Cu^{2+} and 25 µM Zn^{2+}	25 µM Zn^{2+} Vs 100 µM Ni^{2+} and 25 µM Zn^{2+}	100 µM Zn^{2+} Vs 100 µM Cu^{2+} and 100 µM Zn^{2+}	100 µM Zn^{2+} Vs 100 µM Cu^{2+} and 100 µM Zn^{2+}
−, −, −, −, −	−, −, −, −, −	−, −, −, −, −	−, −, −, −, −
−, −, −, −, −	−, −, −, −, −	−, −, −, −, −	−, −, −, −, −

−: The operator was not able to observe a color difference between the Zn^{2+} standard solutions (25 and 100 µM) in the absence of and in the presence of 100 µM Cu^{2+} or Ni^{2+}.

Table S7. Interference threshold assessment by analyzing 20 µL Hg^{2+} standard solutions (5, 12.5, 25, 50, 100, 150, 250 and 500 µM) prepared in ultrapure water and using PALL Biodyne A and Whatman Grade 1 as porous material. The interference threshold was assessed by comparing the M^{n+} standard solution output with the output obtained by analyzing non-fortified ultrapure water. The comparison was performed by 5 operators.

		**M^{n+} (µM)**							
	**Comparison**	0 vs 5	0 vs 12.5	0 vs 25	0 vs 50	0 vs 100	0 vs 150	0 vs 250	0 vs 500
Hg^{2+}	**PALL Biodyne A**	−, −, −, −, −	−, −, −, −, −	−, −, −, −, −	−, −, −, −, −	−, −, +, −, −	+, +, +, +, +	+, +, +, +, +	+, +, +, +, +
	**Whatman Grade 1**	−, −, −, −, −	−, −, −, −, −	−, −, −, −, −	−, −, −, −, −	−, −, −, −, −	−, −, +, −, −	+, +, +, +, +	+, +, +, +, +

−: The operator was not able to observe a color difference between the two M^{n+} standard solutions.
+: The operator was able to observe a color difference between the two M^{n+} standard solutions.

Table S8. Shelf-life evaluation at 25 and 37°C up to 2 months. The color assignment for the analysis of fortified ultrapure water (20 µL of 12.5 µM and 50 µM or 20 µL of 25 µM and 100 µM Zn^{2+}) using PALL Biodyne A and Whatman Grade 1 as porous material was performed by 5 operators in three replicates.

			Time (days)				
	**T (°C)**	**Zn^{2+} (µM)**	0	7	15	30	60
**PALL Biodyne A**	25	12.5	LO, LO, LO, LO, LO	LO, LO, LO, LO, LO	LO, LO, LO, LO, LO	LO, LO, LO, LO, LO	LO, LO, LO, LO, LO
		50	R, LR, R, R, R	R, LR, R, R, R	R, R, R, R, R	R, R, R, R, R	R, LR, R, R, R
	37	12.5	LO, LO, LO, LO, LO	LO, LO, LO, LO, LO	O, O, O, O, O	LO, LO, LO, LO, LO	LO, LO, LO, LO, LO
		50	R, R, R, R, R	R, R, R, R, R	R, R, R, R, R	R, R, R, R, R	R, R, R, R, R
**Whatman Grade 1**	25	25	O, O, O, O, O	O, O, O, O, O	O, O, O, O, O	O, O, O, O, O	O, O, DO, O, DO
		100	R, R, R, R, R	R, R, R, R, R	R, R, R, R, R	R, R, R, R, R	R, R, R, R, R
	37	25	O, O, O, O, O	O, O, O, O, O	O, O, O, O, O	O, O, O, O, O	O, O, DO, O, DO
		100	R, R, R, R, R	R, R, R, R, R	R, R, R, R, R	R, R, LR, R, R	R, R, R, R, R

LO: light orange, O: orange; DO: dark orange; LR: light red; R: red.

Table S9. Shelf-life evaluation at 25 and 37°C up to 2 months. The ability of the device to distinguish Zn^{2+} concentrations above and below the cut-off (20 µL of 12.5 µM vs 50 µM or 20 µL of 25 µM vs 100 µM Zn^{2+}, prepared in ultrapure water), using PALL Biodyne A and Whatman Grade 1 as porous material, was qualitatively evaluated by 5 operators in three replicates.

			**Time (days)**				
	**T (°C)**	**Zn^{2+} (µM)**	0	7	15	30	60
**PALL Biodyne A**	25	12.5 vs 50	+, +, +, +, +	+, +, +, +, +	+, +, +, +, +	+, +, +, +, +	+, +, +, +, +
	37	12.5 vs 50	+, +, +, +, +	+, +, +, +, +	+, +, +, +, +	+, +, +, +, +	+, +, +, +, +
**Whatman Grade 1**	25	25 vs 100	+, +, +, +, +	+, +, +, +, +	+, +, +, +, +	+, +, +, +, +	+, +, +, +, +
	37	25 vs 100	+, +, +, +, +	+, +, +, +, +	+, +, +, +, +	+, +, +, +, +	+, +, +, +, +

+: The operator was able to observe a color difference between the two Zn^{2+} concentrations.

Table S10. Results for the analysis of fortified ultrapure water (20 µL of 25 and 100 µM Zn^{2+}) using Whatman Grade 1 as porous material. Qualitative evaluation was performed by 5 operators in ten replicates.

	Zn^{2+} (µM)	
	25	100
**Color assessment**	O, O, O, O, O	R, R, R, R, R
**Sample classification based on the cut-off threshold**	N, N, N, N, N	P, P, P, P, P

O: orange; R: red.
N: negative; P: positive.

Table S11. Results for the analysis of fortified tap water (20 µL of 0, 25 and 100 µM Zn^{2+}) using Whatman Grade 1 as porous material. Qualitative evaluation was performed by 5 operators in three replicates.

	**Zn^{2+} (µM)**		
	0	25	100
**Color assessment**	DY, DY, DY, DY, DY	O, O, O, O, O	R, R, R, R, R
**Sample classification based on the cut-off threshold**	N, N, N, N, N	N, N, N, N, N	P, P, P, P, P

DY: dark yellow; O: orange; R: red.
N: negative; P: positive.

Table S12. Results for the analysis of fortified urine (20 µL of 0, 12.5, 25, 50, 100 and 500 µM Zn^{2+}) using PALL Biodyne A as porous material. Qualitative evaluation was performed by 5 operators in three replicates.

**Zn^{2+} (µM)**					
0	12.5	25	50	100	500
DY, DY, DY, DY, DY	LO, O, O, LO, O	O, DO, DO, O, DO	R, R, R, LR, R	DR, F, DR, DR, F	F, F, F, F, F

DY: dark yellow; LO: light orange, O: orange; DO: dark orange; LR: light red; R: red; DR: dark red; F: fuchsia.

Table S13. Assessment of reading time effect (1, 5, 10 and 30 min) by analyzing Zn^{2+} standard solutions (20 µL of 12.5 and 50 µM) prepared in urine sample (0.76 µM Zn^{2+}) and using PALL Biodyne A as porous material. Qualitative evaluation was performed by 5 operators in three replicates.

		Time (minutes)			
		1	5	10	30
**Zn^{2+} (µM)**	12.5	LO, O, O, O, O	O, O, O, O, O	O, O, O, O, O	O, O, O, O, O
	50	DO, R, R, R, R	R, R, R, R, R	R, R, R, R, R	R, R, R, R, R

LO: light orange, O: orange; DO: dark orange; R: red.

Table S14. Assessment of color variation at different readout times (1, 5, 10 and 30 min) by analyzing Zn^{2+} standard solutions (20 µL of 12.5 and 50 µM) prepared in urine sample (0.76 µM Zn^{2+}). The ability of the device to distinguish the two Zn^{2+} concentrations, using PALL Biodyne A as porous material, was qualitatively evaluated by 5 operators in three replicates.

		**Time (minutes)**			
		1	5	10	30
**Zn^{2+} (µM)**	12.5 vs 50	+, +, +, +, +	+, +, +, +, +	+, +, +, +, +	+, +, +, +, +

+: The operator was able to observe a color difference between the two Zn^{2+} concentrations.

Table S15. Results for the analysis of patients' urinary samples using PALL Biodyne A as porous material. Qualitative evaluation was performed by 5 operators in three replicates.

		**Color assessment**	**Sample classification based on the cut-off threshold**
**Sample**	**A1**	R, R, R, R, R	N, N, N, N, N
	**A2**	R, R, R, R, R	N, N, N, N, N
	**A3**	R, R, R, R, R	N, N, N, N, N
	**A4**	O, O, O, O, O	P, P, P, P, P
	**A5**	R, R, R, R, R	N, N, N, N, N
	**A6**	F, F, F, R, R	N, N, N, N, N
	**A7**	F, F, R, R, R	N, N, N, N, N
	**A8**	F, F, F, R, R	N, N, N, N, N
	**A9**	DO, R, R, R, R	P, N, N, N, N
	**A10**	DY, DY, DY, DY, DY	P, P, P, P, P
	**A11**	LO, LO, LO, LO, LO	P, P, P, P, P
	**A12**	O, O, O, LO, O	P, P, P, P, P
	**A13**	O, LO, O, O, LO	P, P, P, P, P
	**A14**	DY, LO, DY, DY, LO	P, P, P, P, P
	**A15**	DY, DY, DY, DY, LO	P, P, P, P, P
	**A16**	LO, LO, LO, LO, LO	P, P, P, P, P
	**A17**	DY, DY, DY, LO, DY	P, P, P, P, P

DY: dark yellow; LO: light orange; O: orange; DO: dark orange; R: red; F: fuchsia.
N: negative; P: positive.
